# Characteristics and adverse outcomes of Chinese adolescent pregnancies between 2012 and 2019

**DOI:** 10.1038/s41598-021-92037-x

**Published:** 2021-06-15

**Authors:** Yanxia Xie, Xiaodong Wang, Yi Mu, Zheng Liu, Yanping Wang, Xiaohong Li, Li Dai, Qi Li, Mingrong Li, Peiran Chen, Jun Zhu, Juan Liang

**Affiliations:** 1 National Office for Maternal and Child Health Surveillance of China, West China Second University Hospital, Sichuan University, Ren Min South Road Section 3 No.17, Chengdu, Sichuan China; 2 Department of Obstetrics, West China Second University Hospital, Sichuan University, Ren Min South Road Section 3 No.17, Chengdu, Sichuan China; 3 Key Laboratory of Birth Defects and Related Diseases of Women and Children (Sichuan University), Ministry of Education, Chengdu, China; 4 Medical Big Data Center, Sichuan University, Chengdu, Sichuan China

**Keywords:** Epidemiology, Health care

## Abstract

We aimed to describe the characteristics of adolescent pregnancy, determine its effect on adverse maternal and perinatal outcomes and explore whether that association varies with gestational age with the goal of proposing specific recommendations for adolescent health in China. This study included 2,366,559 women aged 10–24 years who had singleton pregnancies between 2012 and 2019 at 438 hospitals. Adolescent pregnancy was defined as younger than 20 years of age. We used multivariable logistic regression to estimate the effects. Women aged 20–24 years served as the reference group in all analyses. The proportion of rural girls with adolescent pregnancies rebounded after 2015 even though common-law marriage in rural areas decreased. Higher risks of eclampsia (adjusted odds ratio (aOR) 1.87, 95% confidence interval (CI) 1.57 ~ 2.23), severe anaemia (aOR 1.18, 95% CI 1.09 ~ 1.28), maternal near miss (MNM; aOR 1.24, 95% CI 1.12 ~ 1.37), and small for gestational age (SGA; aOR 1.30, 95% CI 1.28 ~ 1.33) were observed when gestational age was > 37 weeks. Adolescent pregnancy was independently associated with increased risks of other perinatal outcomes. Further implementation of pregnancy prevention strategies and improved health care interventions are needed to reduce adolescent pregnancies and prevent adverse fertility outcomes among adolescent women in China at a time when adolescent fertility rate is rebounding.

## Introduction

Adolescent pregnancy usually refers to intended or unintended pregnancy in women aged of 10–19 years^1^. Babies born to adolescent mothers account for approximately 11% of all births worldwide, with 90% occurring in low-income countries^2^. Some important factors have influenced adolescent pregnancy in recent decades in China, such as the marriage market, common-law marriage, and unprotected sex^3–5^. A recent study indicated that although China’s total fertility rate remains far less than the replacement-level fertility, the age-specific fertility rate for the 15- to 19-year age group rebounded from 6.0 births per 1000 in 2000 to 9.2 births per 1000 in 2015^5^. Adolescent pregnancy contributes to maternal mortality, perinatal and infant mortality, and the vicious cycle of ill health and poverty^2^. It has a long-term impact on the physical and mental health of adolescents and continues to be a challenging public health issue in China and some low-income countries.

Studies from high- or low-income countries have consistently reported that adolescent pregnancies are at increased risk for some adverse maternal and perinatal outcomes, such as eclampsia^6,7^, puerperal endometritis^6^, stillbirth^8^, low birth weight (LBW)^9,10^, preterm delivery^8–11^, small for gestational age (SGA)^10^, and intra-hospital early neonatal death^6,8^. In addition, although Hebei Province reported an adolescent pregnancy effect on some adverse maternal and perinatal outcomes^8^, this study was a partial analysis conducted prior to 2017, and the data from only one province were inadequately representative of China. To date, no studies have reported data representing all of China. Furthermore, gestational age is the major determinant of maternal and neonatal health; adolescents are still in the process of puberty growth and development, and they are not physically ready for pregnancy. With an increase in gestational age, the harm to adolescents' bodies is greater than that of adults, but their bearing capacity is limited; even in the same gestational age category, adolescent pregnancy suffers a higher risk of adverse outcomes than adults, but the association between the range of gestational age and the risk of adolescent pregnancies has rarely been investigated^12,13^.

In this research, based on China’s National Maternal Near Miss Surveillance System (NMNMSS), data were collected from 2012 to 2019 covering 30 provinces of mainland China. The objective of this large hospital-based study was to describe the distribution of adolescent pregnancy, to determine the adolescent pregnancy effect on adverse maternal and perinatal outcomes and to explore whether that association varies with gestational age with the goal of proposing specific recommendations for adolescent health in China.

## Methods

### Data sources

Individual maternal data were collected through the NMNMSS from 1 January 2012 to 31 December 2019. The NMNMSS was established in 2010 and covers 441 member hospitals that manage more than 1000 deliveries annually. The member hospitals are located in 326 districts or counties throughout 30 provinces in mainland China, excluding Tibet. The NMNMSS collects socio-demographic and obstetric information on pregnant and postpartum women from the obstetric departments of surveillance hospitals. The collected data included the name and code of the hospital, date of delivery, number of antenatal visits, maternal educational and marital status, mother's date of birth, gestational age at delivery or at termination of pregnancy, mode of delivery, parity, and number of foetuses. The detailed sample methods have been described elsewhere^14–16^, and the data provided to us were de-identified.

The data used in our study are not publicly available, and all methods were performed in accordance with the relevant guidelines and regulations.

### Definition

All women aged 10–24 years who had singleton pregnancies delivered in 438 hospitals (3 hospitals were excluded because data were not reported since 2012) between 1 January 2012 and 31 December 2019 in the NMNMSS were included in the present study. We excluded women who had multiple births for the following reasons. First, multiple births accounted for only 1.1% of all births in adolescent mothers of the present study, with a relatively small sample size. Second, the inherent increased risk of multiple births with adverse maternal outcomes is increased for a variety of mixed causes and confounding factors, such as genetic factors, use of ovulation-inducting drugs, and assisted reproduction, but we have not collected data on these variables from the NMNMSS. Third, some previous studies on the relationship between certain exposures and pregnancy outcomes were also limited to single births, to better explore the relationship between the two. Therefore, we have limited our study to single-birth pregnancies, which we believe helped us to better explore the more common and representative association between adolescent pregnancy and adverse pregnancy outcomes.

Maternal age was defined as the age of the mother in completed years at the time of delivery. Our main exposure was being less than 20 years of age at the time of delivery or termination of pregnancy. Since the vast majority of adolescent pregnant women were 18–19 years old, women were categorized into 3 groups according to maternal age: under 18, 18–19, and 20–24 years. Women aged 20–24 years had the lowest risk of adverse outcomes, and they served as the reference group in all analyses.

Maternal outcomes included maternal near miss (MNM) and some of the most common maternal complications in China, including antepartum haemorrhage, postpartum haemorrhage, preeclampsia, eclampsia, HELLP syndrome, gestational diabetes mellitus (GDM), premature rupture of membranes (PROM) and severe anaemia (haemoglobin concentration < 70 g/L, with the definition excluding postpartum haemorrhage). The definitions and criteria of maternal death and MNM were fully consistent with the WHO recommendations^17^. Antepartum haemorrhage, including uterine rupture, placenta previa, placenta accreta, abruptio placenta or other types, occurred during or before the second stage of labour. Postpartum haemorrhage, including soft birth canal lacerations, uterine atony, retained placenta or other types, was defined as obstetric haemorrhage greater than 500 ml during vaginal delivery or 1,000 ml during caesarean section and occurred in or after the third stage of labour^18^.

Perinatal outcomes were restricted to live births at 28 gestational weeks or later or to a birthweight of more than 1000 g, in accordance with the definition of third-trimester stillbirth from the WHO^19^. Gestational age in China is generally ascertained on the basis of the last menstrual period or the ultrasound examination when the date of the last menstrual period is unknown^20^. Gestational age was categorized into 3 groups: < 28 w, 28–36 w, and >  = 37 w. Perinatal outcomes of interest in this study were stillbirth, preterm birth (less than 37 gestational weeks), SGA (birth weight less than the 10th percentile for gestational age^21^), and low birth weight (less than 2,500 g).

Four modes of delivery were defined: miscarriage or abortion, termination of pregnancy (TOP), vaginal delivery, and caesarean section. Miscarriage or abortion was defined as loss at a gestational age less than 28 weeks and included miscarriage and induced abortion. TOP referred to induced labour at more than 28 weeks of gestation due to foetal or maternal causes.

The usual definitions of region, marital status, number of antenatal care visits, maternal educational status, caesarean history and parity were used, as detailed elsewhere. Based on the hospitals’ locations, we classified regions as east urban, east rural, central urban, central rural, west urban, and west rural. The hospital level was defined based on a comprehensive standard that includes the number of beds in the hospital and the medical staff, clinical department categories, type and quantity of medical equipment and funding of the hospital^20^. Level 1 represents the smallest hospitals, and level 3 represents the largest hospitals.

### Statistical analysis

Over the 8-year period, 12,184,452 pregnancies were recorded in our database, and we excluded data for 9,182,421 mothers older than 24 years, 30,866 multiple pregnancies, 563,584 for whom maternal age was missing, and 41,022 for whom information on the number of foetuses was missing. The remaining 2,366,559 pregnant women constituted the study population, of whom 283,746 were adolescents. Overall, adolescents accounted for 2.5% of all deliveries in our database. The subject inclusion process is presented in Additional Fig. 1.

We first described the distribution of demographic characteristics, prenatal care, gestational age and delivery outcome by maternal age group. The incidence of adverse maternal outcomes and perinatal outcomes was calculated for each maternal age group. The Maternal mortality ratio (MMR) during hospitalization was defined as the number of maternal deaths per 100,000 live births. The incidence of other adverse maternal outcomes was defined as the cases per 1,000 pregnancies. The incidence of adverse perinatal outcomes (except stillbirth) was defined as the cases per 1,000 live births, while, the incidence of stillbirth was defined as the cases per 1,000 births. The *P* values for trends were determined by logistic regression.

The crude odds ratios (ORs) and adjusted ORs (aORs) along with 95% confidence intervals (CIs) associated with adolescent pregnancies, with reference to the 20- to 24-year-old group, were derived through logistic regression models with adjustment for potential confounders. Potential confounding variables considered for adjustment in the regression models included the clustering of births within hospitals and by region, birth location (urban/rural), hospital level, and year, as well as the mother’s education status, marital status, caesarean history and parity. We did not adjust for intermediate factors (birth weight, small uterine volume, cervical length, and gestational age) in order to estimate the total effect of adolescent pregnancies on risk of adverse pregnancy outcomes. Confounder selection is illustrated in a directed acyclic graph (Additional Fig. 2), which was conducted by an online drawing system (http://www.dagitty.net/dags.html). To explore whether the association risk of adolescent pregnancies varies between different gestational ages, we additionally conducted a subgroup analysis (< 28 w, 28–36 w, and >  = 37 w). The results of the subgroup analysis are detailed further in Additional File 1.

A mediation model was constructed to determine whether the risk for adverse outcomes in adolescent pregnancies was mediated by gestational age. In this model, adolescent pregnancy was a predictor, gestational age was a mediator, and adverse pregnancy outcomes were the outcome. The mediation model involved three models: Model Y = β_Tot_ X (β_Tot_ = total effect), Model M = β_1_ X (β_1_ = indirect effect 1), and Model Y = β_2_ M + β_Dir_ X (β_2_ = indirect effect 2, β_Dir_ = direct effect). The proportion of the mediation effect was calculated using the formula: Mediation effect (%) =  (β_1_ × β_2_/ β_Tot_) × 100%. All statistical calculations were performed using Stata software, version 16.0 (Stata Corp LP., College Station, United States of America). A 2-sided *P* value of less than 0.05 was considered statistically significant. Because we tested multiple types of adverse maternal and perinatal outcomes and several sub-analyses, we conservatively corrected for multiple testing using the Bonferroni correction and controlled for the type I error rate at 0.05/45 = 0.001.

### Ethics approval and consent to participate

Ethical approval for the NMNMSS was provided by the Ethics Committee of West China Second University Hospital, Sichuan University, China (protocol ID, 2012008). Informed consent from the patient was waived from the Ethics Committee, as the data used in this study were obtained from a national routine surveillance system established by the government. Data use was authorized by the National Health Commission, and the data provided to us were de-identified.

## Results

### Characteristics of adolescent pregnancy

**Figure 1:**
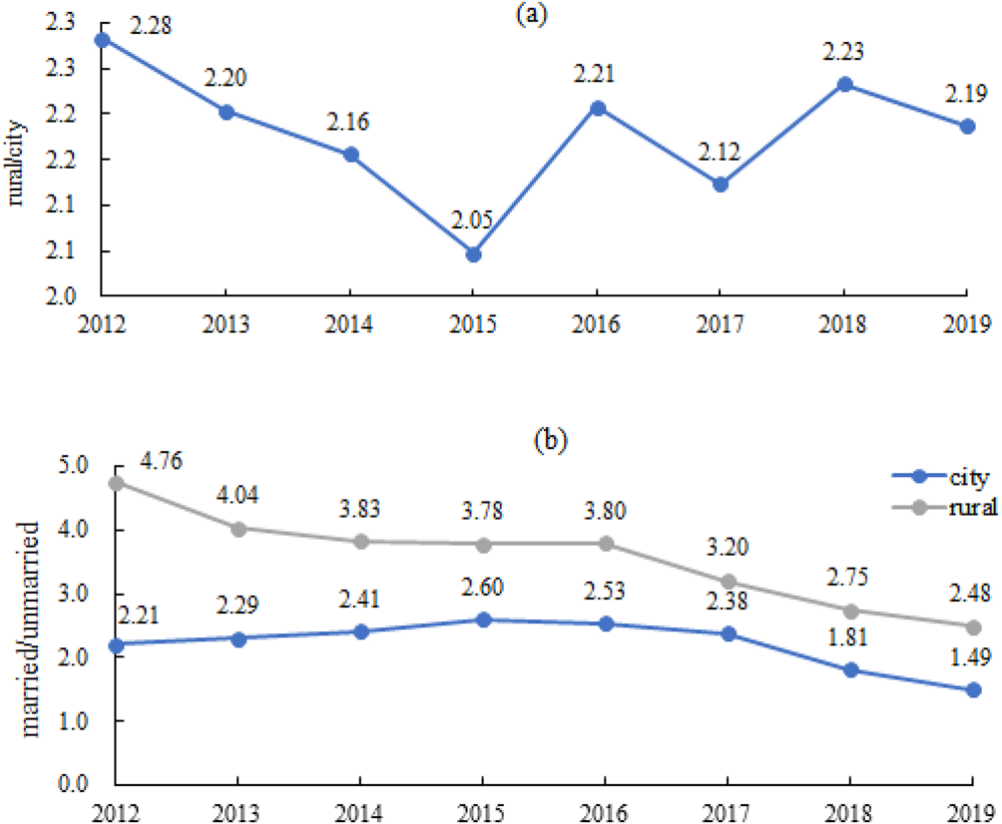
Changes in adolescent pregnancies in the ratio of rural to urban areas (**a**) and ratio of married women to unmarried women (**b**) in 2012–2019.

There were 2,974 (0.1%), 7,847 (0.3%), 20,824 (0.9%), 44,462 (1.9%), 79,214 (3.4%), 128,425 (5.4%), and 2,082,813 (88.0%) pregnancies women aged <  = 14, 15, 16, 17, 18, 19 and 20–24 years, respectively. Adolescent pregnancy was most common in the economically underdeveloped western region (45.0%), followed by the central region (34.1%); it was least common in the eastern region (20.9%), which had the highest level of economic development. In each region, the distribution was larger in rural areas than in urban areas. A total of 68.53% of adolescent pregnancies were from rural areas. The proportion of rural girls with adolescent pregnancies rebounded after 2015 (Fig. 1a). The prevalence of common-law marriage in rural areas have decreased among adolescent pregnancies, but was still more common than that in urban areas. In both urban and rural areas, the proportion of unmarried adolescent pregnancies continued to increase in 2012–2019, and childbearing among unmarried women was more common in urban areas (Fig. 1b). Moreover, compared with women aged 20–24 years, adolescent mothers were more likely to be unmarried and nulliparous and to have a lower educational level or inadequate prenatal care during pregnancy (Table 1).

**Table 1:** Maternal characteristics and delivery information in relation to maternal age (< 25 years old) in China, 2012–2019.

	All adolescents	Maternal age (y)						
		< = 14	15	16	17	18	19	20–24
**Total pregnancies, n**	283,746	2,974	7,847	20,824	44,462	79,214	128,425	2,082,813
**Region, n (%)**								
East urban	17,995 (6.3%)	164 (5.5%)	431 (5.5%)	1,183 (5.7%)	2539 (5.7%)	4,935 (6.2%)	8,743 (6.8%)	238,761 (11.5%)
East rural	41,341 (14.6%)	390 (13.1%)	1,126 (14.4%)	3,088 (14.8%)	6484 (14.6%)	11,717 (14.8%)	18,536 (14.4%)	294,914 (14.2%)
Central urban	26,184 (9.2%)	380 (12.8%)	710 (9.1%)	1,810 (8.7%)	3739 (8.4%)	6,967 (8.8%)	12,578 (9.8%)	315,245 (15.1%)
Central rural	70,690 (24.9%)	616 (20.7%)	1,642 (20.9%)	4,403 (21.1%)	10,297 (23.2%)	19,676 (24.8%)	34,056 (26.5%)	578,921 (27.8%)
West urban	45,117 (15.9%)	554 (18.6%)	1,305 (16.6%)	3,350 (16.1%)	7234 (16.3%)	12,717 (16.1%)	19,957 (15.5%)	312,875 (15.0%)
West rural	82,419 (29.1%)	870 (29.3%)	2,633 (33.6%)	6,990 (33.6%)	14,169 (31.8%)	23,202 (29.3%)	34,555 (26.9%)	342,097 (16.4%)
**Antenatal care, n (%)**								
None	17,623 (6.2%)	483 (16.2%)	999 (12.7%)	2,052 (9.9%)	3,293 (7.4%)	4,781 (6.0%)	6,015 (4.7%)	53,966 (2.6%)
1 ~ 3	52,863 (18.6%)	855 (28.8%)	2,077 (26.5%)	4,969 (23.9%)	920 (21.0%)	14,584 (18.4%)	21,058 (16.4%)	217,842 (10.5%)
4 ~ 6	114,560 (40.4%)	904 (30.4%)	2,727 (34.8%)	7,757 (37.3%)	17,631 (39.7%)	32,338 (40.8%)	53,203 (41.4%)	765,950 (36.8%)
7 ~ 9	61,673 (21.7%)	443 (14.9%)	1,276 (16.3%)	3,829 (18.4%)	9,013 (20.3%)	17,311 (21.9%)	29,801 (23.2%)	573,165 (27.5%)
> = 10	29,383 (10.4%)	186 (6.3%)	538 (6.9%)	1,629 (7.8%)	3,967 (8.9%)	8,043 (10.2%)	15,020 (11.7%)	416,833 (20.0%)
Missing	7,644 (2.7%)	103 (3.5%)	230 (2.9%)	588 (2.8%)	1,238 (2.8%)	2,157 (2.7%)	3,328 (2.6%)	55,057 (2.6%)
**Education, n (%)**								
College or higher	10,377 (3.7%)	0 (0.0%)	0 (0.00%)	0 (0.0%)	1,265 (2.9%)	2,886 (3.6%)	6,226 (4.9%)	378,031 (18.2%)
High school	60,933 (21.5%)	292 (9.8%)	1,080 (13.8%)	3,213 (15.4%)	7,891 (17.8%)	16,749 (21.1%)	31,708 (24.7%)	650,070 (31.2%)
Middle school	185,198 (65.3%)	1,793 (60.3%)	5,191 (66.2%)	14,063 (67.5%)	30,511 (68.6%)	52,617 (66.4%)	81,023 (63.1%)	959,872 (46.1%)
Primary school	21,933 (7.7%)	592 (19.9%)	1,233 (15.7%)	2,619 (12.6%)	4,083 (9.2%)	5,776 (7.3%)	7,630 (5.9%)	63,671 (3.1%)
Illiteracy	2,362 (0.8%)	48 (1.6%)	92 (1.2%)	217 (1.0%)	416 (0.9%)	640 (0.8%)	949 (0.7%)	9,029 (0.4%)
Missing	29,43 (1.0%)	249 (8.4%)	251 (3.2%)	712 (3.4%)	296 (0.7%)	546 (0.7%)	889 (0.7%)	22,140 (1.1%)
**Marital status, n (%)**								
Unmarried	68,678 (24.2%)	2,974 (100.0%)	2,926 (37.3%)	6,911 (33.2%)	12,768 (28.7%)	18,680 (23.6%)	24,419 (19.0%)	54,822 (2.6%)
Married	215,018 (75.8%)	0 (0.0%)	4,919 (62.7%)	13,908 (66.8%)	31,689 (71.3%)	60,514 (76.4%)	103,988 (81.0%)	2,027,591 (97.4%)
Missing	50 (0.02%)	0 (0.0%)	2 (0.0%)	5 (0.0%)	5 (0.0%)	20 (0.0%)	18 (0.0%)	400 (0.0%)
**Parity, n (%)**								
Nulliparous	248,334 (87.5%)	2,717 (91.4%)	7,354 (93.7%)	19,448 (93.4%)	40,559 (91.2%)	69,623 (87.9%)	108,633 (84.6%)	1,556,810 (74.8%)
Multiparous without scar	29,474 (10.4%)	200 (6.7%)	404 (5.2%)	1,140 (5.5%)	3,285 (7.4%)	8,055 (10.2%)	16,390 (12.8%)	393,713 (18.9%)
Multiparous with scar	5,670 (2.0%)	54 (1.8%)	79 (1.0%)	216 (1.0%)	576 (1.3%)	1,461 (1.8%)	3,284 (2.6%)	129,944 (6.2%)
Missing	268 (0.1%)	3 (0.1%)	10 (0.1%)	20 (0.1%)	42 (0.1%)	75 (0.1%)	118 (0.1%)	2,346 (0.1%)
**Gestational weeks, n (%)**								
< 28	24,772 (8.8%)	700 (23.9%)	1,430 (18.5%)	2,905 (14.1%)	4,533 (10.3%)	6,644 (8.5%)	8,560 (6.7%)	78,241 (3.8%)
28–36	23,433 (8.3%)	411 (14.1%)	993 (12.8%)	2,235 (10.9%)	4,190 (9.5%)	6,384 (8.1%)	9,220 (7.2%)	116,556 (5.6%)
> = 37	232,887 (82.1%)	1,815 (61.0%)	5,318 (67.8%)	15,411 (74.0%)	35,230 (79.2%)	65,470 (82.7%)	109,643 (85.4%)	1,878,372 (90.2%)
Missing	2,654 (0.9%)	48 (1.6%)	106 (1.4%)	273 (1.3%)	509 (1.1%)	716 (0.9%)	1,002 (0.8%)	9,644 (0.5%)
**Delivery mode, n (%)**								
Vaginal delivery	188,392 (66.4%)	1,655 (55.7%)	4,854 (61.9%)	13,613 (65.4%)	29,823 (67.1%)	53,220 (67.2%)	85,227 (66.4%)	1,294,789 (62.2%)
Caesarean section	68,329 (24.1%)	517 (17.4%)	1,374 (17.5%)	3,974 (19.1%)	9,659 (21.7%)	18,846 (23.8%)	33,959 (26.4%)	702,918 (33.8%)

The incidence of adverse maternal and perinatal outcomes, such as miscarriage (4.4 per 1,000 pregnancies), abortion (41.0 per 1,000 pregnancies), TOP (4.9 per 1,000 pregnancies), puerperal infection (1.3 per 1,000 pregnancies), eclampsia (1.3 per 1,000 pregnancies), HELLP syndrome (0.5 per 1,000 pregnancies), severe anaemia (6.4 per 1,000 pregnancies), MNM (4.2 per 1,000 pregnancies), stillbirth (20.4 per 1,000 births), preterm delivery (77.9 per 1,000 live births), LBW (77.3 per 1,000 live births), SGA (154.5 per 1,000 live births), and early neonatal death (2.3 per 1,000 live births) was higher in adolescent pregnancies. These outcomes consistently increased with decreasing maternal age and were consistently highest among mothers under 15 years of age (coefficient < 0 and *P* < 0.05). In addition, abortion was more common in adolescent pregnancies. However, the incidence of antepartum haemorrhage, postpartum haemorrhage, GDM, and PROM increased as maternal age increased (coefficient > 0 and *P* < 0.05) (Table 2).

**Table 2:** Incidence of adverse maternal and perinatal outcome according to maternal age in China, 2012–2019**.**

	All adolescents	Maternal age (y)							Coef	*p* vaule
		< = 14	15	16	17	18	19	20–24		
**Maternal outcomes, cases (incidence, per 1,000 pregnancies)**										
Miscarriage	1,244 (4.4)	18 (6.1)	39 (5.0)	128 (6.1)	202 (4.5)	349 (4.4)	508 (4.0)	5,411 (2.6)	− 0.20	< 0.001
Abortion	11,638 (41.0)	267 (89.8)	588 (74.9)	1237 (59.4)	2,080 (46.8)	3,264 (41.2)	4,202 (32.7)	37,229 (17.9)	− 0.33	< 0.001
TOP	1,949 (6.9)	98 (33.0)	166 (21.2)	282 (13.5)	368 (8.3)	465 (5.9)	570 (4.4)	5,796 (2.8)	− 0.40	< 0.001
Antepartum hemorrhage	2,204 (7.8)	22 (7.4)	57 (7.3)	199 (9.6)	357 (8.0)	613 (7.7)	956 (7.4)	19,404 (9.3)	0.06	< 0.001
Postpartum hemorrhage	9,164 (32.3)	92 (30.9)	263 (33.5)	663 (31.8)	1,437 (32.3)	2,540 (32.1)	4,169 (32.5)	71,594 (34.4)	0.02	< 0.001
Puerperal infection	362 (1.3)	5 (1.7)	11 (1.4)	25 (1.2)	49 (1.1)	98 (1.2)	174 (1.4)	2,221 (1.1)	− 0.06	0.019
Preeclampsia	4824 (17.0)	31 (10.4)	158 (20.1)	339 (16.3)	735 (16.5)	1,331 (16.8)	2,230 (17.4)	35,932 (17.3)	0.01	0.135
Eclampsia	382 (1.3)	5 (1.7)	21 (2.7)	41 (2.0)	77 (1.7)	114 (1.4)	124 (1.0)	1,136 (0.5)	− 0.35	< 0.001
HELLP syndrome	128 (0.5)	0 (0)	5 (0.6)	5 (0.2)	28 (0.6)	38 (0.5)	52 (0.4)	726 (0.3)	− 0.10	0.013
GDM	2,784 (9.8)	16 (5.4)	56 (7.1)	177 (8.5)	365 (8.2)	753 (9.5)	1,417 (11.0)	50,177 (24.1)	0.42	< 0.001
PROM	19,159 (67.5)	125 (42.0)	417 (53.1)	1,232 (59.2)	2,862 (64.4)	5,351 (67.6)	9,172 (71.4)	175,503 (84.3)	0.11	< 0.001
Severe anemia	1,813 (6.4)	13 (4.4)	62 (7.9)	139 (6.7)	340 (7.6)	481 (6.1)	778 (6.1)	9,003 (4.3)	− 0.15	< 0.001
MNM	1,183 (4.2)	16 (5.4)	39 (5.0)	109 (5.2)	218 (4.9)	329 (4.2)	472 (3.7)	6,107 (2.9)	− 0.15	< 0.001
MMR*, per 100,000 live births	23 (9.0)	0 (0)	0 (0)	1 (5.8)	6 (15.4)	7 (9.8)	9 (7.6)	154 (7.7)	− 0.03	0.76
**Perinatal outcomes, cases (incidence, per 1,000 live births)**										
LBW	19,645 (77.3)	224 (107.3)	649 (107.2)	1,771 (102.7)	3,460 (88.7)	5,641 (79.1)	7,900 (66.8)	93,459 (47.0)	− 0.22	< 0.001
Preterm delivery	19,784 (77.9)	253 (121.2)	688 (113.7)	1,712 (99.3)	3,506 (89.9)	5,493 (77.0)	8,132 (68.7)	106,335 (53.5)	− 0.18	< 0.001
Stillbirth	5,300 (20.4)	219 (94.9)	392 (60.8)	733 (40.8)	974 (24.4)	1,344 (18.5)	1,638 (13.7)	15,664 (7.8)	− 0.42	< 0.001
SGA	39,241 (154.5)	344 (164.8)	1,149 (189.8)	3,128 (181.4)	6,620 (169.8)	11,261 (157.8)	16,739 (141.5)	200,472 (100.8)	− 0.20	< 0.001
Early neonatal death	584 (2.3)	20 (9.6)	25 (4.1)	57 (3.3)	126 (3.2)	134 (1.9)	222 (1.9)	2,393 (1.2)	− 0.29	< 0.001

*TOP* terminate of pregnancy; TOP refers to induced labor at more than 28 weeks of gestation due to fetal or maternal causes.

*GDM* gestational diabetes mellitus, *PROM* premature rupture of membranes, *MMR* maternal mortality ration, *MNM* maternal near miss, *LBW* low birth weight, *SGA* small-for-gestation-age.

*MMR refers to the maternal mortality ratio during hospitalization.

### Effect of adolescent pregnancy on adverse maternal and perinatal outcomes

The associations between adolescent pregnancies and adverse maternal and perinatal outcomes are shown in Table 3. The results here were based on the Bonferroni correction. Compared with mothers aged 20–24 years, adolescent mothers had higher risks of TOP (aOR 1.74, 95% CI 1.56 ~ 1.93), eclampsia (aOR 1.70, 95% CI 1.49 ~ 1.94), HELLP syndrome (aOR 1.28, 95% CI 1.04 ~ 1.58), severe anaemia (aOR 1.16, 95% CI 1.09 ~ 1.24), MNM (aOR 1.16, 95% CI 1.07 ~ 1.27), stillbirth (aOR 1.63, 95% CI 1.52 ~ 1.75), preterm delivery (aOR 1.41, 95% CI 1.36 ~ 1.47), LBW (aOR 1.44, 95% CI 1.39 ~ 1.50), SGA (aOR 1.28, 95% CI 1.25 ~ 1.30), and early neonatal death (aOR 1.40, 95% CI 1.24 ~ 1.59). Younger mothers faced higher risks of TOP, eclampsia, and severe anaemia. However, the risks of GDM (aOR 0.60, 95% CI 0.56 ~ 0.65), PROM (aOR 0.78, 95% CI 0.75 ~ 0.82), and maternal death during hospitalization (aOR 0.47, 95% CI 0.30 ~ 0.73) were significantly lower in adolescent mothers than in mothers 20–24 years old. In addition, the youngest mothers faced lower risks of GDM, and PROM.

**Table 3:** Association between adolescent pregnancies and adverse maternal and perinatal outcome in China, 2012–2019.

	Case/Num		Adolescents								
			< 18			18–19			Total		
	Adolescents	20–24	Crude OR*	Adjusted OR**	*P* value^#^	Crude OR*	Adjusted OR**	*P* value^#^	Crude OR*	Adjusted OR**	*P* value^#^
**Maternal outcomes**											
Miscarriage	1,244/283,746	5,411/2,082,813	1.96 (1.70 ~ 2.26)	0.85 (0.71 ~ 1.02)	0.074	1.59 (1.46 ~ 1.73)	0.96 (0.87 ~ 1.06)	0.427	1.69 (1.56 ~ 1.84)	0.93 (0.83 ~ 1.03)	0.175
Abortion	11,638/283,746	37,229/2,082,813	3.19 (2.74 ~ 3.71)	0.99 (0.81 ~ 1.22)	0.954	2.05 (1.84 ~ 2.28)	1.01 (0.90 ~ 1.14)	0.86	2.35 (2.09 ~ 2.64)	1.01 (0.87 ~ 1.16)	0.94
TOP	1,949/283,746	5,796/2,082,813	4.36 (3.21 ~ 5.90)	2.57 (2.19 ~ 3.01)	** < 0.001**	1.80 (1.43 ~ 2.25)	1.38 (1.23 ~ 1.55)	** < 0.001**	2.48 (1.92 ~ 3.20)	1.74 (1.56 ~ 1.93)	** < 0.001**
Antepartum hemorrhage	2,204/28,3746	19,404/2,082,813	0.89 (0.78 ~ 1.03)	0.96 (0.85 ~ 1.08)	0.509	0.81 (0.74 ~ 0.88)	0.90 (0.84 ~ 0.96)	0.002	0.83 (0.76 ~ 0.91)	0.92 (0.85 ~ 0.98)	0.012
Postpartum hemorrhage	9,164/283,746	72,594/2,082,813	0.94 (0.85 ~ 1.03)	0.91 (0.83 ~ 1.01)	0.064	0.94 (0.88 ~ 0.99)	0.93 (0.88 ~ 0.99)	0.034	0.94 (0.88 ~ 1.00)	0.93 (0.87 ~ 0.99)	0.033
Puerperal infection	362/283,746	2,221/2,082,813	1.11 (0.85 ~ 1.44)	1.00 (0.78 ~ 1.27)	0.998	1.23 (1.03 ~ 1.47)	1.22 (1.03 ~ 1.44)	0.021	1.20 (0.99 ~ 1.43)	1.16 (0.99 ~ 1.36)	0.074
Preeclampsia	4,824/283,746	35,932/2,082,813	0.96 (0.87 ~ 1.06)	0.88 (0.81 ~ 0.95)	0.002	0.99 (0.93 ~ 1.06)	0.94 (0.90 ~ 0.98)	0.008	0.99 (0.92 ~ 1.05)	0.92 (0.88 ~ 0.97)	0.001
Eclampsia	382/283,746	1,136/2,082,813	3.47 (2.73 ~ 4.42)	2.06 (1.67 ~ 2.54)	** < 0.001**	2.10 (1.79 ~ 2.47)	1.56 (1.34 ~ 1.82)	** < 0.001**	2.47 (2.12 ~ 2.88)	1.70 (1.49 ~ 1.94)	** < 0.001**
HELLP syndrome	128/283,746	726/2,082,813	1.43 (1.01 ~ 2.03)	1.29 (0.89 ~ 1.88)	0.179	1.24 (0.99 ~ 1.55)	1.27 (1.01 ~ 1.62)	0.046	1.29 (1.07 ~ 1.56)	1.28 (1.04 ~ 1.58)	** < 0.001**
GDM	2,784/283,746	50,177/2,082,813	0.33 (0.28 ~ 0.39)	0.54 (0.48 ~ 0.60)	** < 0.001**	0.43 (0.38 ~ 0.48)	0.62 (0.58 ~ 0.67)	** < 0.001**	0.40 (0.36 ~ 0.45)	0.60 (0.56 ~ 0.65)	** < 0.001**
PROM	19,159/283,746	175,503/2,082,813	0.70 (0.66 ~ 0.75)	0.69 (0.64 ~ 0.74)	** < 0.001**	0.82 (0.78 ~ 0.86)	0.82 (0.78 ~ 0.85)	** < 0.001**	0.79 (0.75 ~ 0.83)	0.78 (0.75 ~ 0.82)	** < 0.001**
Severe anemia	1,813/283,746	9,003/2,082,813	1.69 (1.49 ~ 1.91)	1.20 (1.08 ~ 1.34)	**0.001**	1.41 (1.30 ~ 1.51)	1.15 (1.07 ~ 1.23)	** < 0.001**	1.48 (1.37 ~ 1.60)	1.16 (1.09 ~ 1.24)	** < 0.001**
MNM	1183/283,746	6,107/2,082,813	1.72 (1.48 ~ 1.99)	1.22 (1.05 ~ 1.41)	0.008	1.32 (1.20 ~ 1.45)	1.14 (1.05 ~ 1.25)	0.003	1.42 (1.29 ~ 1.57)	1.16 (1.07 ~ 1.27)	**0.001**
Maternal death^##^	23/283,746	154/2,082,813	1.24 (0.55 ~ 2.83)	0.36 (0.14 ~ 0.94)	0.036	1.04 (0.65 ~ 1.67)	0.53 (0.33 ~ 0.84)	0.006	1.10 (0.74 ~ 1.62)	0.47 (0.30 ~ 0.73)	**0.001**
**Perinatal outcomes**											
Stillbirth	5,300/283,746	15,664/2,082,813	4.57 (4.04 ~ 5.17)	2.35 (2.12 ~ 2.61)	** < 0.001**	2.00 (1.84 ~ 2.17)	1.38 (1.29 ~ 1.47)	** < 0.001**	2.65 (2.42 ~ 2.90)	1.63 (1.52 ~ 1.75)	** < 0.001**
Premature delivery	19,784/283,746	106,335/2,082,813	1.87 (1.75 ~ 2.00)	1.67 (1.58 ~ 1.77)	** < 0.001**	1.37 (1.31 ~ 1.44)	1.33 (1.28 ~ 1.38)	** < 0.001**	1.50 (1.42 ~ 1.57)	1.41 (1.36 ~ 1.47)	** < 0.001**
LBW	19,645/283,746	93,459/2,082,813	2.12 (2.01 ~ 2.24)	1.63 (1.55 ~ 1.72)	** < 0.001**	1.56 (1.49 ~ 1.63)	1.38 (1.33 ~ 1.43)	** < 0.001**	1.70 (1.63 ~ 1.78)	1.44 (1.39 ~ 1.50)	** < 0.001**
SGA	39,241/283,746	200,472/2,082,813	1.89 (1.81 ~ 1.97)	1.34 (1.29 ~ 1.38)	** < 0.001**	1.55 (1.50 ~ 1.59)	1.26 (1.23 ~ 1.28)	** < 0.001**	1.63 (1.58 ~ 1.68)	1.28 (1.25 ~ 1.30)	** < 0.001**
Early neonatal death	584/283,746	2393/2,082,813	2.95 (2.53 ~ 3.45)	1.92 (1.60 ~ 2.30)	** < 0.001**	1.56 (1.39 ~ 1.75)	1.23 (1.08 ~ 1.40)	0.002	1.91 (1.73 ~ 2.12)	1.40 (1.24 ~ 1.59)	** < 0.001**

*TOP* terminate of pregnancy; TOP refers to induced labor at more than 28 weeks of gestation due to fetal or maternal causes.

*GDM* gestational diabetes mellitus, *PROM* premature rupture of membranes, *MNM* maternal near miss, *LBW* low birth weight, *SGA* small-for-gestation-age.

*Adjusted for clustering of births within hospitals.

**Adjusted for clustering of births within hospitals, region, birth location (urban/rural), hospital level, year, and the mother’s education status, marital status, caesarean history and parity.

^#^*P* value of the adjusted model.

^#^^#^Maternal death refers to death during hospitalization.

As shown in Table 4, the mediation effect of gestational age on the association between adolescent pregnancy and adverse outcomes was estimated at 66.9% of antepartum haemorrhage, 52.8% of postpartum haemorrhage, 33.2% of stillbirth, 33.0% of MNM, 26.7% of early neonatal death, 10.8% of puerperal infection, 8.0% of PROM, 6.0% of eclampsia, and 4.0% of GDM and severe anaemia.

**Table 4:** Mediation effect of gestational age on the adolescent pregnancy- adverse pregnancy outcome association.

	X-M		M-Y		X–Y		Mediation effect,%
	β_1_	*P*	β_2_	*P*	β_3_	*P*	
**Maternal outcomes**							
Antepartum hemorrhage	− 0.077	< 0.001	− 0.045	< 0.001	− 0.009	< 0.001	66.9%
Postpartum hemorrhage	− 0.077	< 0.001	0.028	< 0.001	− 0.002	< 0.001	52.8%
Puerperal infection	− 0.077	< 0.001	0.003	< 0.001	0.002	0.002	10.8%
Preeclampsia	− 0.077	< 0.001	− 0.006	< 0.001	− 0.001	0.102	−
Eclampsia	− 0.077	< 0.001	− 0.007	< 0.001	0.01	< 0.001	6.0%
HELLP syndrome	− 0.077	< 0.001	− 0.009	< 0.001	0.001	0.127	−
GDM	− 0.077	< 0.001	0.016	< 0.001	− 0.03	< 0.001	4.0%
PROM	− 0.077	< 0.001	0.02	< 0.001	− 0.018	< 0.001	8.0%
Severe anemia	− 0.077	< 0.001	− 0.005	< 0.001	0.009	< 0.001	4.0%
MNM	− 0.077	< 0.001	− 0.03	< 0.001	0.005	< 0.001	33.0%
Maternal death during hospitalization	− 0.077	< 0.001	− 0.007	< 0.001	− 0.001	0.826	−
**Perinatal outcomes**							
Stillbirth	− 0.038	< 0.001	− 0.368	< 0.001	0.028	< 0.001	33.2%
LBW	− 0.021	< 0.001	− 0.478	< 0.001	0.034	< 0.001	23.3%
SGA	− 0.021	< 0.001	− 0.028	< 0.001	0.055	< 0.001	1.1%
Early neonatal death	− 0.021	< 0.001	− 0.116	< 0.001	0.007	< 0.001	26.7%

*GDM* gestational diabetes mellitus, *PROM* premature rupture of membranes, *MNM* maternal near miss, *LBW* low birth weight, *SGA* small-for-gestation-age.

**Figure 2:**
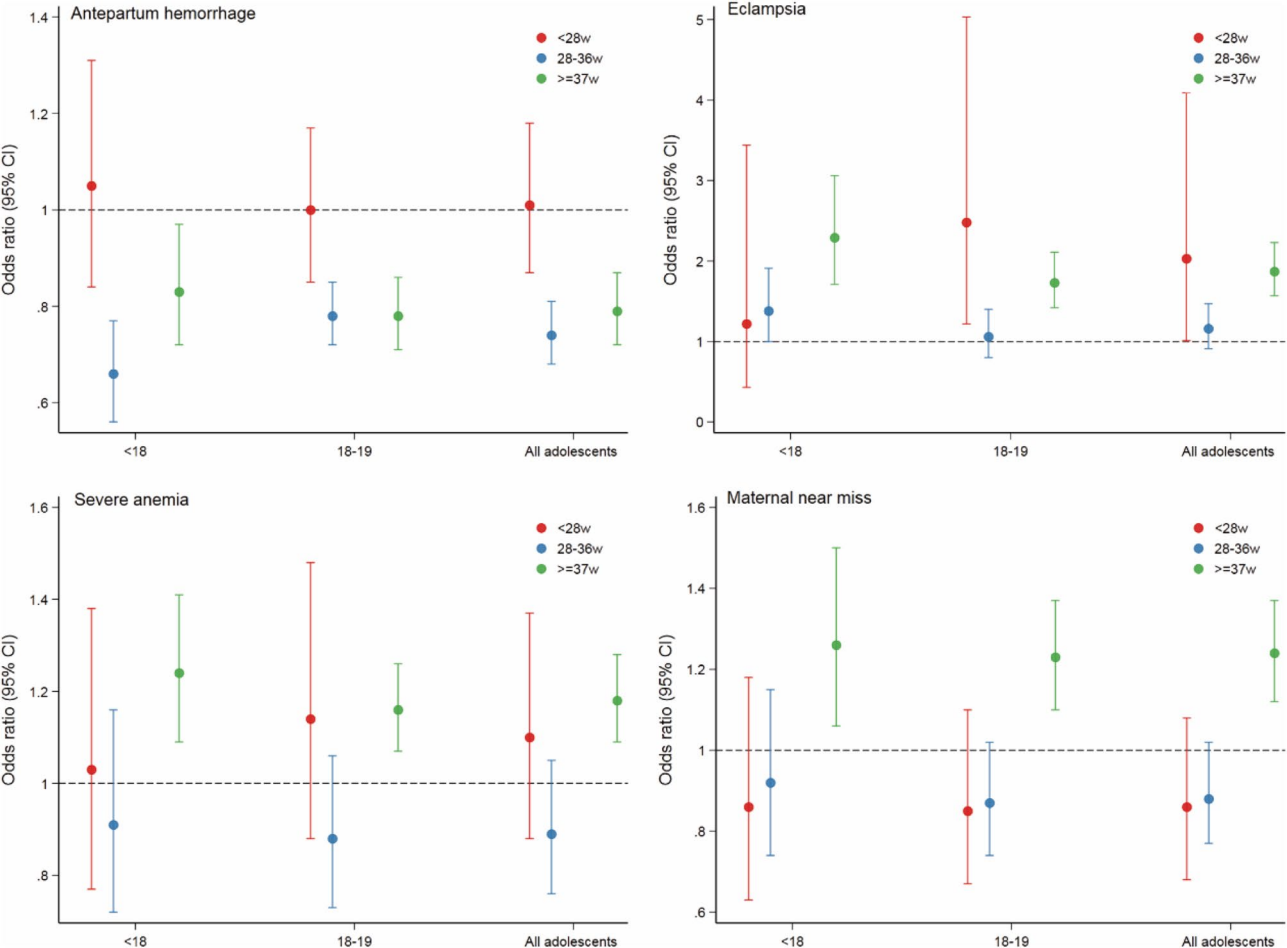
Adjusted odds ratios and 95% confidence intervals of adolescent pregnancies for adverse maternal outcomes stratified by gestational age, women aged 20–24 years served as the reference group.

**Figure 3:**
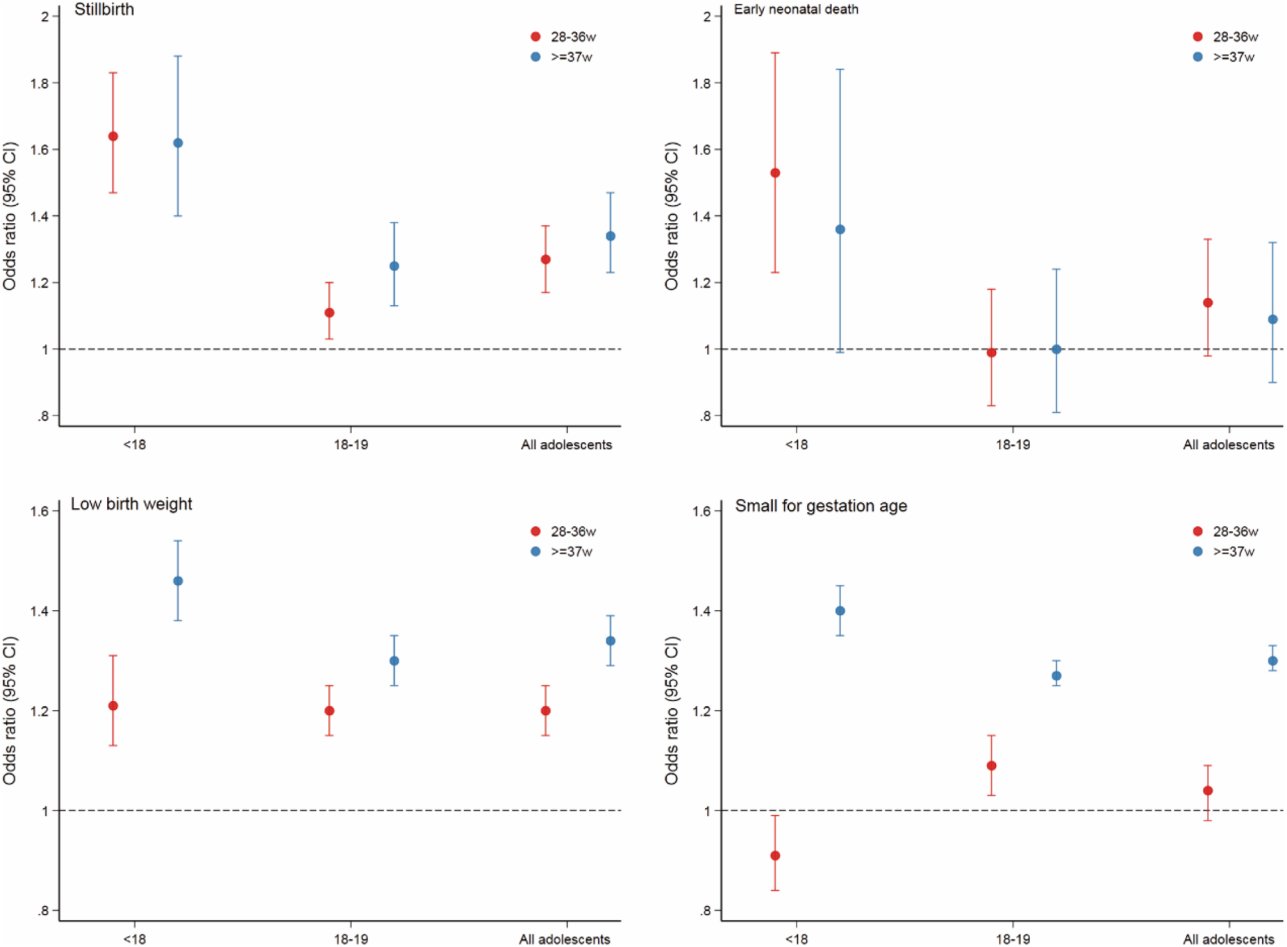
Adjusted odds ratios and 95% confidence intervals of adolescent pregnancies for adverse perinatal outcomes stratified by gestational age, women aged 20–24 years served as the reference group.

Furthermore, in the sub-analyses, we found that the risk of antepartum haemorrhage was significantly lower in adolescent mothers when the gestational age was 28–36 weeks (aOR 0.74, 95% CI 0.68 ~ 0.81) or >  = 37 weeks (aOR 0.79, 95% CI 0.72 ~ 0.87). The same results were found when a subgroup of adolescents 18–19 years old was considered. Compared with mothers aged 20 to 24 years, adolescent mothers had higher risks of eclampsia (aOR 1.87, 95% CI 1.57 ~ 2.23), severe anaemia (aOR 1.18, 95% CI 1.09 ~ 1.28), and MNM (aOR 1.24, 95% CI 1.12 ~ 1.37) when the gestational age was greater than 37 weeks; the youngest mothers faced higher risks, whereas mothers aged 18 to 19 had smaller increases in risks (Fig. 2, Additional Table 1). Moreover, adolescent mothers were at greater risks of stillbirth (28–36 w: aOR 1.27, 95% CI 1.17 ~ 1.37; <  = 37 w: aOR 1.34, 95% CI 1.23 ~ 1.47) and LBW (28–36 w: aOR 1.20, 95% CI 1.15 ~ 1.25; <  = 37 w: aOR 1.34, 95% CI 1.29 ~ 1.39) regardless of gestational age; the younger their age, the greater the risks were. Similar results were found for the risk of SGA (aOR 1.30, 95% CI 1.28 ~ 1.33) among adolescent mothers when gestational age was greater than 37 weeks. Furthermore, at 28–36 weeks of gestation, adolescent mothers younger than 18 years old had an increased risk of intra-hospital early neonatal death (aOR 1.53, 95% CI 1.23 ~ 1.89) (Fig. 3, Additional Table 2). The results here were based on the Bonferroni correction.

## Discussion

The main findings of our study were that adolescent pregnancy occurred mainly in economically underdeveloped areas and rural areas (68.5%), especially in western rural areas (29.1%). The proportion of rural girls with adolescent pregnancies rebounded after 2015, and the proportion of unmarried people increased year by year. The adolescent pregnancy effect on some adverse maternal and perinatal outcomes varied with gestational age. Higher risks of eclampsia, severe anaemia, and MNM were observed at a gestational age greater than 37 weeks, and lower risks of antepartum haemorrhage and postpartum haemorrhage were observed at gestational age >  = 28 weeks among adolescent mothers compared with mothers aged 20–24 years. Our results further showed that adolescent pregnancy was independently associated with increased risks of stillbirth, preterm delivery, LBW, and SGA and an increased risk of intra-hospital early neonatal death among non-adult mothers when the gestational age was 28–36 weeks. In addition, we observed higher risks of adverse perinatal outcomes with decreasing maternal age as well as TOP, eclampsia, and severe anaemia.

Early marriage and adolescent fertility rates were included in the 12 key indicators of adolescent health and well-being proposed by the 2016 Lancet Commission on Adolescent Health and Wellbeing^22^. The new "Marriage Law of the People's Republic of China" promulgated in 1981 stipulated that the minimum marriage age for women was 20 years old, which, to a certain extent, restricted the occurrence of early marriage and childbearing behaviours among Chinese female adolescents. The open-door policy and economic reforms of the 1980s not only injected vitality into economic development but also considerably changed social norms and values pertaining to love and marriage. Attitudes towards sex have become more open, while the negative consequences of early sexual intercourse have become issues of health and social concern^23,24^. There are inherent differences between urban and rural areas; for example, the overall economic levels, education and social customs (such as, the educational concept of “son preference” in some backward areas, or parents’ traditional thought of “early marriage and early childbearing”) are poorer or more prudent in rural areas than in urban areas^23^. The data in our study showed that the proportion of the common-law marriage decreased from 2012 to 2019, but it continued to occur among adolescents, especially in areas with low levels of economic development, such as rural areas. The proportion of unmarried adolescent pregnancies increased year by year in both urban and rural areas, and childbearing outside of marriage was more common in urban areas.

For some adolescent women, pregnancy and childbirth are planned and wanted, but for many others, they are not. Several factors contribute to unplanned and unwanted pregnancies in adolescents. Over the past 20 years, sexual activity outside marriage and high-risk sexual behaviours have increased among Chinese adolescents, contributing to more unintended pregnancies^25^. The proportion of unmarried adolescent pregnancies in our study increased from 21.5% in 2012 to 32.3% in 2019. In addition, a survey of adolescent sexual and reproductive health in China showed that 21.4% of unmarried young women aged 15–24 years did not use contraception during their most recent episode of intercourse^5^. Some do not know how to avoid pregnancy, while others are unable to obtain condoms and contraceptives. Adolescents may be unable to refuse unwanted sex or to resist coerced sex^26^. Even in the context of reform and opening up, conservative taboos about sex in China are still deeply rooted, especially in rural areas. Sex education and female self-protection awareness are lacking^27^. In addition, society maintains a contemptuous attitude towards unmarried pregnancy; coupled with the age limit of the marriage law and the custom of early marriage and early childbearing in rural areas, this contributes to the existence of common-law marriage. In our study, the majority of adolescent pregnancies are likely to be from common-law marriages in rural areas. These adolescents may be under pressure to marry and to bear children early, and they may have limited educational and employment prospects. Unmarried pregnant adolescents, mainly in urban areas (urban 22.2% vs. rural 16.5%), often use abortion or induction of labour to terminate their pregnancy. Abortion is more common in adolescent pregnancies than miscarriage is, and the miscarriage, abortion or TOP rates of adolescent pregnancies increased from 9.2% to 13.4% during our study period (2012–2019). Failure to complete school, lack of occupation, lack of income and social prejudice might explain why adolescents have higher rates of miscarriage or abortion and TOP, and the youngest mothers (< 18 years old) face the highest risks of TOP compared with the risk for mothers aged 20 to 24 years. Moreover, adolescents who become pregnant are less likely than adults to be able to obtain legal and safe abortions to terminate their pregnancies.

Adolescents, especially young adolescents, are still in the process of puberty growth and development. They do not have the ability to live independently, and they are not physically or psychologically ready for pregnancy. Premature pregnancy has adverse effects on health^1,2,6–8,10,11,19,22,28–30^. Our study demonstrated that higher risks of eclampsia, severe anaemia, and MNM were observed among adolescent mothers when the gestational age was greater than 37 weeks than among mothers aged 20–24 years. Fear of public condemnation and limited access to skilled prenatal care, poor nutritional levels and a lack of prevention and treatment for hypertensive disorders, anaemia, or other complications during the early and second trimesters among adolescents could be reasonable explanations for the high risks of eclampsia, severe anaemia, and MNM in the third trimester. In addition, low caloric intake as well as increased iron requirements for red blood cell expansion during adolescence may contribute to a high risk of severe anaemia among adolescent mothers^31^.

Studies have reported that adolescents aged 15–19 years are twice as likely to die during pregnancy or childbirth as women over 20 years of age, and adolescents under 15 years of age are five times more likely to die during pregnancy or childbirth^10,32^. We found that MMR during hospitalization in adolescent pregnancy was higher than that for mothers aged 20–24 (8.1 per 100,000 pregnancies vs. 7.4 per 100,000 pregnancies). However, adolescent pregnancy was not a risk factor for MMR during hospitalization after adjusting for confounding factors. Moreover, compared with adult pregnancy women (age 20–24 years), adolescent pregnancy was a protective factor for maternal death during hospitalization. The pregnancy risk assessment for pregnant women in China is divided into low risk, general risk, higher risk, highest risk, and pregnant women with infectious diseases. Since young pregnant women (< = 18 years old) are included in the general risk for management during pregnancy and childbirth, effective management measures may be one of the reasons for the reduction in the risk of maternal death. On the other hand, a higher risk of MNM was observed among adolescent mothers. However, a series of rescue systems for MNM have been established by medical institutions in China in recent years. If a critical situation for a pregnant woman occurs, she can be sent to the treatment centre hospital in time through this system; this has effectively prevented most MNM situations from developing into maternal deaths.

In terms of adverse perinatal outcomes, consistent with previous studies^6–8,10,11^, we found that compared with adult pregnant women, adolescent mothers had an increased risk of stillbirth, preterm birth, SGA, and LBW, and the degree of risk decreased with age. The theory that foetuses and mothers compete for nutrition is a common explanation for why infants born to adolescent mothers may be more prone to LBW^1,33^. A surge in leptin in late pregnancy may block fat breakdown, increase the use of glucose for maternal growth, and reduce the energy needed for foetal growth ^33^. In addition, the production of glycine, an amino acid needed for foetal growth and development, may be affected in young mothers^34^. Micronutrient deficiencies may be another biological pathway that may affect foetal growth ^30^. Previous studies have also shown that a short cervix (< = 25 mm) and small uterine volume can increase the risk of preterm birth, in adolescent mothers ^28,35,36^. Furthermore, depression and stress-related pathways may mediate adverse consequences, such as preterm birth (e.g., by stimulating the release of placental corticotropin releasing hormone)^37^.

Previous studies of intra-hospital early neonatal mortality (IHENM) in adolescent pregnancy have yielded conflicting results. A multi-country study suggested that IHENM was not increased in infants born to adolescent mothers compared with infants born to mothers aged 20–24^6^. However, data from Hebei Province reported that the risk of early neonatal death was increased in adolescent mothers compared with mothers who were 25–34 years old^8^. We observed a high risk of IHENM in general among adolescent mothers, but after stratifying according to gestational age, we found a high risk only in adolescents (younger than 18 years of age) whose gestational age was 28–36 weeks. The higher rates of SGA and LBW in adolescent mothers aged < 18 may explain this result.

Our study found higher risks of adverse outcomes among adolescent mothers, suggested that adolescent pregnancy prevention is crucial. First, the government should take measures to further reduce the occurrence of marriage before the legal age. Early marriage (< 18 years) and early sexual debut have been reported to be determinants of adolescent pregnancy^2^. Second, it is of vital importance to reduce teenage pregnancy by conducting a range of adolescent-targeted strategies, including expanding sexuality and health education, building life skills, retaining adolescents in school, reducing coerced sex among adolescents, and providing contraceptive counselling and service, which specifically meet the needs for modern contraception in sexually active unmarried girls^29^. Finally, for pregnant adolescents, it is necessary to reduce the occurrence of unsafe abortion; increase the use of skilled antenatal, childbirth and postnatal care; and offer and promote postpartum and post-abortion contraception through multiple home visits and/or clinic visits to reduce the adverse effects of pregnancy on adolescents’ long-term health and to reduce their chances of second pregnancies.

The data in this study were collected from a large and well-established national registry, providing a reliable overview of the association between adolescent pregnancies and adverse outcomes. Our study is unique in its sample size and representativeness because it includes records from different facilities and regions (approximately 10% of all deliveries in mainland China each year). Furthermore, comprehensive data-quality-control strategies were continually performed to ensure data validity. There are also several limitations in this study. First, the vast majority of adolescent pregnant women were 18–19 years old, and the results were mainly based on older adolescent pregnant women. The estimation of adverse risks could be biased in this study. Second, our monitoring objects in the hospital were all pregnant women admitted to the hospital but did not include those who went only to the outpatient clinic. Adolescent mothers are more likely to seek outpatient services to terminate pregnancy. Third, to explore changes in the characteristics of adolescent pregnancy, the proportion ratio was used instead of the rate. Although the proportion ratio cannot describe the frequency or intensity, it can explain the proportion or distribution of the internal components. Moreover, it is likely that residual confounding exists in our analyses. The database did not contain information about smoking, drinking, weight gain during pregnancy or economic status, all of which might affect adverse pregnancy outcomes.

## Conclusion

Chinese adolescent pregnancy was mainly distributed in economically underdeveloped areas. The majority of adolescent pregnancies were likely to have inadequate prenatal care during pregnancy and poor education levels. The effect of adolescent pregnancies on some adverse maternal and perinatal outcomes varied with gestational age. Higher risks of adverse perinatal outcomes increased with decreasing maternal age, as do partial adverse maternal outcomes. These findings emphasize the importance of further implementation of pregnancy prevention strategies and improved health care interventions to reduce adolescent fertility and to avoid adverse fertility outcomes among adolescent women in China at a time when adolescent fertility rate is rebounding.

## Supplementary Information

Supplementary Information.
