# Supplementary material for: Characteristics and adverse outcomes of Chinese adolescent pregnancies between 2012 and 2019
Source: Sci Rep. 2021 Jun 15;11:12508. doi: 10.1038/s41598-021-92037-x (PMC8206124; doi:10.1038/s41598-021-92037-x)

**Additional Tables**

| **Additional Table 1.** Association between adolescent pregnancies and adverse maternal outcome according to gestational age in China, 2012-2019. | | | | | | | | | | | | |
| --- | --- | --- | --- | --- | --- | --- | --- | --- | --- | --- | --- | --- |
|  | Gestational weeks | Case/Num. | | Adolescents | | | | | | | | |
|  |  |  |  | <18 | | | 18-19 | | | Total | | |
|  |  | Adolescents | 20-24 | Crude OR^*^ | Adjusted OR^**^ | *P* vaule^#^ | Crude OR* | Adjusted OR^**^ | *P* vaule^#^ | Crude OR^*^ | Adjusted OR^**^ | *P* vaule^#^ |
| Antepartum hemorrhage | <28w | 288/24,772 | 1,265/78,241 | 0.70 (0.57~0.86) | 1.05 (0.84~1.31) | 0.689 | 0.72 (0.62~0.84) | 1.00 (0.85~1.17) | 0.981 | 0.72 (0.62~0.82) | 1.01 (0.87~1.18) | 0.85 |
|  | 28-36w | 989/23,433 | 7,391/116,556 | 0.56 (0.47~0.66) | 0.66 (0.56~0.77) | **<0.001** | 0.70 (0.63~0.77) | 0.78 (0.72~0.85) | **<0.001** | 0.65 (0.58~0.73) | 0.74 (0.68~0.81) | **<0.001** |
|  | >=37w | 906/232,887 | 10,618/1,878,372 | 0.73 (0.62~0.85) | 0.83 (0.72~0.97) | 0.019 | 0.67 (0.61~0.75) | 0.78 (0.71~0.86) | **<0.001** | 0.69 (0.62~0.76) | 0.79 (0.72~0.87) | **<0.001** |
| Postpartum hemorrhage | <28w | 267/24,772 | 911/78,241 | 1.01 (0.81~1.28) | 1.26 (0.95~1.67) | 0.107 | 0.87 (0.71~1.06) | 1.04 (0.86~1.27) | 0.679 | 0.92 (0.78~1.10) | 1.12 (0.92~1.36) | 0.251 |
|  | 28-36w | 897/23,433 | 4,663/116,556 | 0.99 (0.84~1.18) | 0.83 (0.73~0.96) | 0.011 | 0.94 (0.82~1.07) | 0.85 (0.77~0.95) | 0.004 | 0.96 (0.84~1.09) | 0.85 (0.77~0.94) | **0.001** |
|  | >=37w | 7,854/232,887 | 65,602/1,878,372 | 0.98 (0.89~1.09) | 0.90 (0.81~1.01) | 0.056 | 0.96 (0.89~1.03) | 0.92 (0.86~0.99) | 0.017 | 0.96 (0.90~1.04) | 0.92 (0.85~0.99) | 0.018 |
| Preeclampsia | <28w | 50/24,772 | 362/78,241 | 0.34 (0.18~0.63) | 0.54 (0.28~1.02) | 0.059 | 0.50 (0.35~0.70) | 0.73 (0.52~1.01) | 0.057 | 0.44 (0.30~0.62) | 0.66 (0.47~0.93) | 0.018 |
|  | 28-36w | 940/23433 | 8121/116,556 | 0.41 (0.34~0.49) | 0.43 (0.37~0.50) | **<0.001** | 0.63 (0.58~0.70) | 0.66 (0.61~0.72) | **<0.001** | 0.56 (0.51~0.61) | 0.59 (0.55~0.64) | **<0.001** |
|  | >=37w | 3,785/232,887 | 27,232/1,878,372 | 1.19 (1.08~1.32) | 1.00 (0.91~1.09) | 0.998 | 1.10 (1.03~1.18) | 0.97 (0.92~1.03) | 0.315 | 1.12 (1.05~1.20) | 0.98 (0.93~1.03) | 0.434 |
| Eclampsia | <28w | 17/24,772 | 32/78,241 | 1.02 (0.38~2.76) | 1.22 (0.43~3.44) | 0.705 | 2.09 (1.10~3.98) | 2.48 (1.22~5.03) | 0.012 | 1.68 (0.90~3.15) | 2.03 (1.01~4.09) | 0.047 |
|  | 28-36w | 139/23,433 | 456/116,556 | 1.90 (1.40~2.58) | 1.38 (1.00~1.91) | 0.047 | 1.33 (1.02~1.72) | 1.06 (0.80~1.40) | 0.705 | 1.52 (1.22~1.89) | 1.16 (0.91~1.47) | 0.232 |
|  | >=37w | 216/232,887 | 620/1,878,372 | 3.99 (2.96~5.37) | 2.29 (1.71~3.06) | **<0.001** | 2.42 (1.97~2.98) | 1.73 (1.42~2.11) | **<0.001** | 2.81 (2.33~3.39) | 1.87 (1.57~2.23) | **<0.001** |
| GDM | <28w | 37/24,772 | 297/78,241 | 0.30 (0.17~0.53) | 0.48 (0.28~0.83) | 0.009 | 0.45 (0.30~0.68) | 0.65 (0.44~0.95) | 0.025 | 0.39 (0.27~0.56) | 0.59 (0.41~0.83) | 0.003 |
|  | 28-36w | 320/23,433 | 4,269/116,556 | 0.26 (0.20~0.34) | 0.35 (0.27~0.45) | **<0.001** | 0.42 (0.35~0.49) | 0.53 (0.46~0.62) | **<0.001** | 0.36 (0.31~0.43) | 0.48 (0.41~0.55) | **<0.001** |
|  | >=37w | 2,420/232,887 | 45,533/1,878,372 | 0.37 (0.31~0.44) | 0.57 (0.51~0.65) | **<0.001** | 0.44 (0.39~0.49) | 0.63 (0.58~0.67) | **<0.001** | 0.42 (0.37~0.48) | 0.61 (0.57~0.66) | **<0.001** |
| PROM | <28w | 126/24,772 | 771/78,241 | 0.58 (0.40~0.83) | 1.03 (0.70~1.51) | 0.883 | 0.47 (0.36~0.62) | 0.72 (0.56~0.91) | 0.007 | 0.51 (0.40~0.66) | 0.82 (0.65~1.04) | 0.098 |
|  | 28-36w | 3,283/23,433 | 22,707/116,556 | 0.55 (0.50~0.61) | 0.56 (0.51~0.62) | **<0.001** | 0.74 (0.69~0.80) | 0.75 (0.71~0.81) | **<0.001** | 0.67 (0.63~0.72) | 0.69 (0.65~0.74) | **<0.001** |
|  | >=37w | 15,620/232,887 | 151,608/1,878,372 | 0.76 (0.71~0.81) | 0.67 (0.63~0.72) | **<0.001** | 0.84 (0.80~0.88) | 0.79 (0.76~0.82) | **<0.001** | 0.82 (0.78~0.86) | 0.76 (0.73~0.80) | **<0.001** |
| Severe anemia | <28w | 129/24,772 | 417/78,241 | 0.92 (0.68~1.24) | 1.03 (0.77~1.38) | 0.856 | 1.01 (0.79~1.30) | 1.14 (0.88~1.48) | 0.333 | 0.98 (0.80~1.20) | 1.10 (0.88~1.37) | 0.407 |
|  | 28-36w | 223/23,433 | 906/116,556 | 1.42 (.14~1.76) | 0.91 (0.72~1.16) | 0.462 | 1.13 (0.95~1.35) | 0.88 (0.73~1.06) | 0.173 | 1.23 (1.07~1.41) | 0.89 (0.76~1.05) | 0.159 |
|  | >=37w | 1,423/232,887 | 7,599/1,878,372 | 1.73 (1.50~1.99) | 1.24 (1.09~1.41) | **0.001** | 1.44 (1.32~1.57) | 1.16 (1.07~1.26) | **<0.001** | 1.51 (1.39~1.65) | 1.18 (1.09~1.28) | **<0.001** |
| MNM | <28w | 145/24,772 | 600/78,241 | 0.78 (0.59~1.02) | 0.86 (0.63~1.18) | 0.362 | 0.75 (0.61~0.94) | 0.85 (0.67~1.10) | 0.216 | 0.76 (0.63~0.92) | 0.86 (0.68~1.08) | 0.187 |
|  | 28-36w | 320/23,433 | 1,610/116,556 | 1.09 (0.88~1.36) | 0.92 (0.74~1.15) | 0.468 | 0.94 (0.80~1.10) | 0.87 (0.74~1.02) | 0.079 | 0.99(0.86~1.14) | 0.88 (0.77~1.02) | 0.083 |
|  | >=37w | 675/23,2887 | 3,758/1,878,372 | 1.65 (1.38~1.99) | 1.26 (1.06~1.50) | **0.001** | 1.38 (1.23~1.55) | 1.23 (1.10~1.37) | **<0.001** | 1.45 (1.30~1.62) | 1.24 (1.12~1.37) | **<0.001** |
| OR, odd ratio; GDM, gestational diabetes mellitus; PROM, premature rupture of membranes; MNM, maternal near miss. ^*^Adjusted for clustering of births within hospitals. ^**^Adjusted for clustering of births within hospitals, region, birth location(urban/rural), hospital level, year, and the mother’s education status, marital status, caesarean history and parity. ^#^ *P* value of the adjusted model. | | | | | | | | | | | | |

| **Additional Table 2.** Association between adolescent pregnancies and adverse perinatal outcome according to gestational age in China, 2012-2019. | | | | | | | | | | | | | | |  |
| --- | --- | --- | --- | --- | --- | --- | --- | --- | --- | --- | --- | --- | --- | --- | --- |
|  | Gestational weeks | Case/Num. | | Adolescents | | | | | | | | | | |  |
|  |  |  |  | <18 | | | 18-19 | | | | Total | | | |  |
|  |  | Adolescents | 20-24 | Crude OR^*^ | Adjusted OR^**^ | *P* value ^#^ | | Crude OR^*^ | Adjusted OR^**^ | *P* value *#* | | Crude odds ratio^*^ | Adjusted odds ratio^**^ | P value ^#^ | |
| Stillbirth | 28-36w | 3,465/23,055 | 9,916/115,539 | 2.72 (2.40~3.09) | 1.64 (1.47~1.83) | **<0.001** | | 1.51 (1.38~1.65) | 1.11 (1.03~1.20) | 0.007 | | 1.88 (1.71~2.07) | 1.27 (1.17~1.37) | **<0.001** | |
|  | >=37w | 727/232,814 | 3,372/1,877,937 | 2.35 (2.03~2.71) | 1.62 (1.40~1.88) | **<0.001** | | 1.54 (1.39~1.71) | 1.25 (1.13~1.38) | **<0.001** | | 1.74 (1.59~1.91) | 1.34 (1.23~1.47) | **<0.001** | |
| LBW | 28-36w | 11,095/19,574 | 53,518/105,555 | 1.33 (1.23~1.43) | 1.21 (1.13~1.31) | **<0.001** | | 1.25 (1.18~1.31) | 1.20 (1.15~1.25) | **<0.001** | | 1.27 (1.21~1.34) | 1.20 (1.15~1.25) | **<0.001** | |
|  | >=37w | 8,187/232,078 | 38,908/1,874,504 | 2.10 (1.99~2.22) | 1.46 (1.38~1.54) | **<0.001** | | 1.60 (1.54~1.67) | 1.30 (1.25~1.35) | **<0.001** | | 1.73 (1.66~1.80) | 1.34 (1.29~1.39) | **<0.001** | |
| SGA | 28-36w | 2,952/19,574 | 14,535/105,555 | 1.02 (0.94~1.11) | 0.91 (0.84~0.99) | 0.044 | | 1.15 (1.09~1.22) | 1.09 (1.03~1.15) | 0.004 | | 1.11 (1.05~1.17) | 1.04 (0.98~1.09) | 0.193 | |
|  | >=37w | 36,289/232,078 | 185,937/1,874,504 | 2.00 (1.91~2.10) | 1.40 (1.35~1.45) | **<0.001** | | 1.58 (1.54~1.63) | 1.27 (1.25~1.30) | **<0.001** | | 1.68 (1.63~1.74) | 1.30 (1.28~1.33) | **<0.001** | |
| Early neonatal death | 28-36w | 355/19,574 | 1,281/105,555 | 2.10 (1.72~2.55) | 1.53 (1.23~1.89) | **<0.001** | | 1.24 (1.06~1.44) | 0.99 (0.83~1.18) | 0.897 | | 1.50 (1.32~1.71) | 1.14 (0.98~1.33) | 0.087 | |
|  | >=37w | 154/232,078 | 944/1,874,504 | 1.76 (1.33~2.33) | 1.36 (0.99~1.84) | 0.05 | | 1.17 (0.96~1.44) | 1.00 (0.81~1.24) | 0.985 | | 1.32 (1.11~1.57) | 1.09 (0.90~1.32) | 0.384 | |
| OR, odd ratio; LBW: low birth weight; SGA: small-for-gestation-age. ^*^Adjusted for clustering of births within hospitals. ^**^Adjusted for clustering of births within hospitals, region, birth location(urban/rural), hospital level, year, and the mother’s education status, marital status, caesarean history and parity. ^#^ *P* value of the adjusted model. | | | | | | | | | | | | | | |  |

**Additional Figures**

**Additional Figure 1** Flow chart of participant recruitment and derivation of the population used in the final analysis

Abbreviation: NMNMSS, China’s National Maternal Near Miss Surveillance System

**Additional Figure 2** Directed acyclic graph (DAG) illustrating confounder selection

Potential confounding variables considered were the clustering of births within hospitals and by region, birth location (urban/rural), hospital level, and year, as well as the mother’s education status, marital status, caesarean history and parity. Since household income, some psychological factors, and planned pregnancy were not collected data from the NMNMSS. We did not adjust for intermediate factors (birth weight, small uterine volume, cervical length, and gestational age) in order to estimate the total effect of adolescent pregnancies on risk of adverse pregnancy outcomes.

**Additional Figure 1**

**
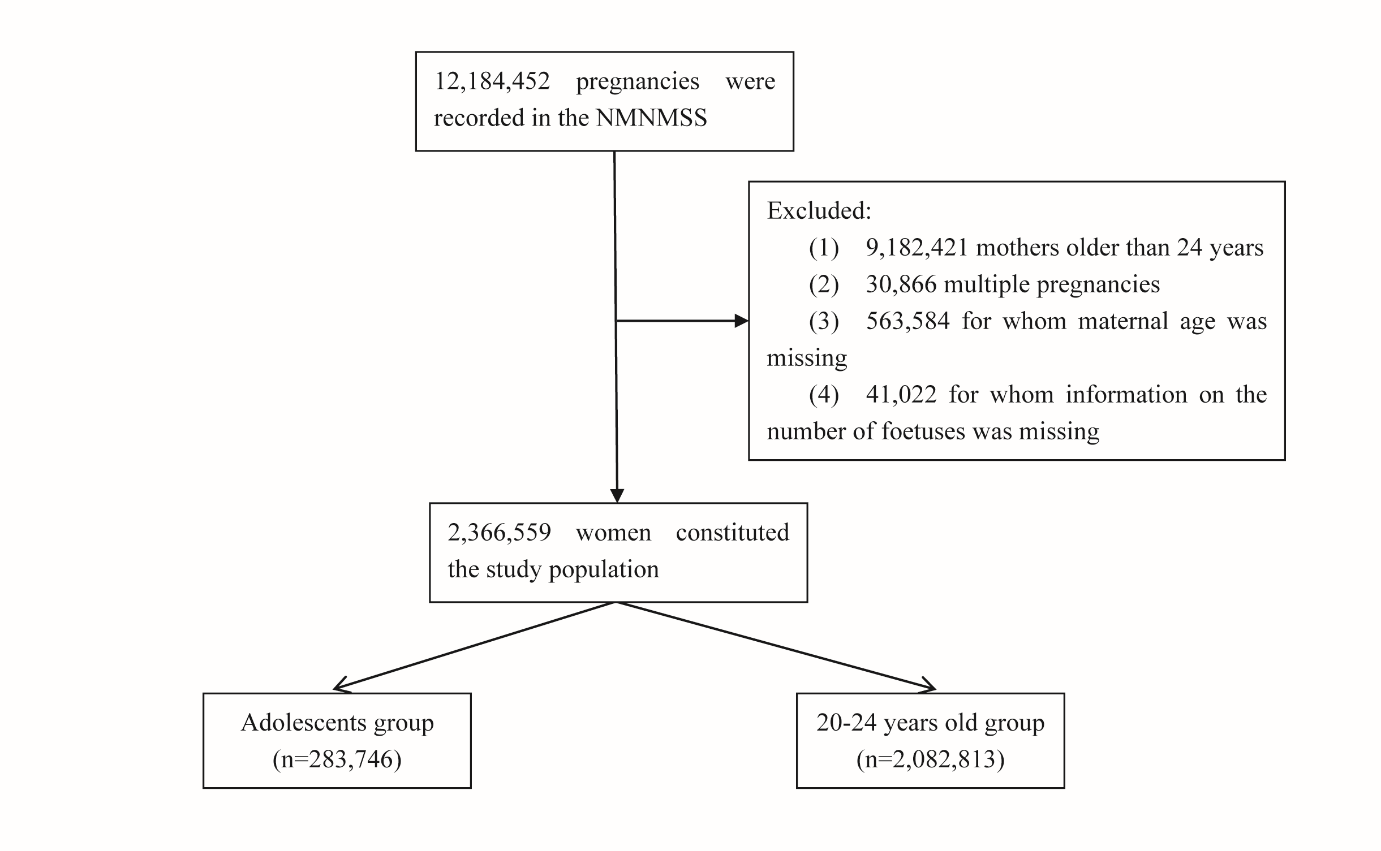
**

**Additional Figure 2**


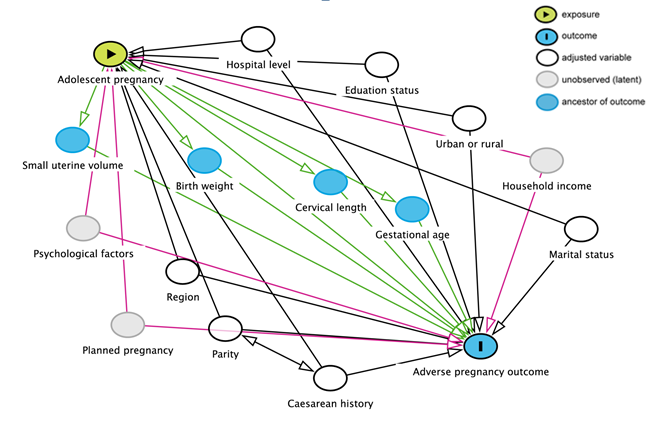

Supplement: Supplementary file 1 — Supplementary Information. [file 41598_2021_92037_MOESM1_ESM.docx]
